# Reversal of Opioid Intoxication in an Infant With Intrauterine Growth Restriction With a Single Dose of Naloxone

**DOI:** 10.7759/cureus.18908

**Published:** 2021-10-19

**Authors:** Utsav Timalsina, Ann Andrasovich, Fernanda E Kupferman, Kusum Viswanathan, Kristina Ericksen

**Affiliations:** 1 Pediatrics, Brookdale University Hospital Medical Center, Brooklyn, USA

**Keywords:** opioid exposure during labor, intrauterine growth restriction, naloxone, hydromorphone, neonatal depression

## Abstract

Perinatal exposure to opioids might result in opioid intoxication in a newborn infant. The routine use of naloxone in an opioid-exposed newborn infant is discouraged due to the risks of precipitating withdrawal and long-term developmental problems associated with naloxone. We describe a case of respiratory and neurological depression in an infant with intrauterine growth restriction (IUGR) following in utero exposure to an opioid two hours before delivery. The infant was apneic with a poor tone immediately after birth. With positive pressure ventilation, the tone and respiratory effort improved, and the baby was admitted to the neonatal intensive care unit (NICU) on oxygen support via nasal cannula. The baby started having bradypnea with shallow breathing and oxygen desaturation at eight hours of life, most likely secondary to intrauterine exposure to hydromorphone which was successfully reversed with a single dose of intravenous naloxone. The infant was discharged on day of life seven with no further symptoms. Naloxone administration might be considered in an IUGR infant with persistent cardiorespiratory and neurological depression who has a history of intrauterine opioid exposure within four hours before delivery provided the mother is not narcotic dependent.

## Introduction

Opioids are being used frequently in high-income countries for labor pain. Estimates suggest that approximately half of women receive opioid analgesia in labor [1]. The most used opioids such as morphine, pethidine, fentanyl, and hydromorphone are highly lipid-soluble and cross the placenta to the fetus when used in pregnant women [2]. Although there are studies on the effects of chronic intrauterine exposure to opioids in newborns due to maternal opioid abuse, there is a paucity of data on the effects of opioid exposure during labor. Neonates with opioid exposure during labor are at high risk of neurological and respiratory depression [2]. The studies comparing the effects of opioid exposure during labor in a normal newborn infant versus an intrauterine growth restriction (IUGR) infant are also limited.

Naloxone is an opioid antagonist that blocks the effects of opiates and reverses neurological and cardiorespiratory depression in opioid-exposed neonates [3]. However, the routine use of naloxone is discouraged in newborn infants due to its long-term adverse effects and the risk of precipitating withdrawal [2]. We describe a case of respiratory and neurological depression in an IUGR baby with intrauterine exposure to hydromorphone successfully reversed by a single dose of naloxone.

## Case presentation

A 2255 g female infant was born to a 31-year-old gravida three, para three (G3P3003) African American mother at 37 weeks and two days gestational age via vaginal delivery. The mother received consistent prenatal care at a tertiary care hospital. The prenatal screening was negative for chlamydia, gonorrhea, syphilis, human immunodeficiency virus, hepatitis B, hepatitis C, cytomegalovirus (CMV), toxoplasma, group B streptococcus, and QuantiFERON gold, rubella immune with no evidence of substance use. Mother was on routine Depo-Provera injection for contraception and tested positive for pregnancy when she came for Depo-Provera injection two months and 22 days after the last injection. Labor was induced at 37 weeks and two days by prostaglandin E2 as the prenatal course was complicated by IUGR. The etiology of IUGR was unknown. The mother denied epidural analgesia for pain management and received 2 mg of intravenous hydromorphone two hours before delivery. The baby was delivered via normal vaginal delivery and received by the neonatal intensive care unit (NICU) team. At birth, the infant was noted to have a heart rate of 130 beats/minute with no respiratory effort and poor tone despite initial stimulation. Positive pressure ventilation was started at 30 seconds of life with pressures of 20/5 and fraction of inspired oxygen (FIO2) of 0.21. The infant improved with resuscitative measures by three minutes of life with appearance, pulse, grimace, activity, and respiration (APGAR) scores of four and eight at one and five minutes, respectively. The baby was transported to the neonatal intensive care unit (NICU) on continuous positive airway pressure (CPAP) with positive end-expiratory pressure (PEEP) of 5 cm H2O and FIO2 of 0.21. Physical examination revealed a birth weight of 2255 g (below the fifth percentile), length of 46 cm (below the fifth percentile), and head circumference of 31.5 cm (below the fifth percentile). Vitals recorded at the time of admission were heart rate (136 beats/minute), respiratory rate (46 breaths/minute), temperature (36.7 degree Celsius), blood pressure (80/52 mm Hg), and oxygen saturation (SPO2) of 94% on oxygen via nasal cannula at 2 L/min with FIO2 of 0.21. The baby was noted to have hypotonia and poor suck. She was started on intravenous fluid containing dextrose 10% at 100 mL/kg/day. Cord blood gas, complete blood count, blood glucose, and bilirubin were within normal limits. At eight hours of life, the baby developed persistent bradypnea and shallow breathing with the respiratory rate in the range of 12 to 16 breaths/minute without any signs of respiratory distress. The heart rate was noted to be decreased from birth in the range of 100 to 110/minute with no associated hypotension or changes in perfusion. There was associated desaturation with SPO2 of 85 % which improved to 91% by increasing the FIO2 to 0.40 at 2 L/min via nasal cannula. Chest X-Ray performed at the time was normal. Due to the concern for respiratory depression secondary to intrauterine exposure to hydromorphone, the decision was made to give intravenous (IV) naloxone. Upon administration of 0.01 mg/kg of IV naloxone, there was an immediate improvement in the respiratory rate to 30 to 40 breaths/minute with improved tone and activity within 30 seconds of giving naloxone. There was no further need for oxygen supplementation and the infant was transitioned to room air. Urine drug screen and blood opioid level were not done. On the day of life two, she continued to do well with no respiratory concerns. However, she continued to have poor suck for which nasogastric tube feeding was initiated. Urine CMV and total immunoglobulin M (IgM) were sent as part of the evaluation for symmetrical IUGR and were negative. Head ultrasound was done due to poor suck and hypotonia which was unremarkable. Intravenous fluid was gradually reduced and discontinued on the day of life four. Nasogastric tube feeding was gradually decreased as oral feeding improved, and the baby achieved full oral feeds with 20 Kcal/oz formula on day of life six. The infant was discharged home on the day of life seven. Parents were instructed to keep strict routine follow-up with the primary care pediatrician.

## Discussion

The management of a newborn infant with cardiorespiratory and neurological depression begins with resuscitative measures. Once the infant is stabilized, understanding the underlying etiologies becomes important. Table 1 describes the common etiologies of cardiorespiratory and neurological depression in a newborn infant [4].

**Table 1:** Etiologies of cardiorespiratory and neurological depression in a neonate

Perinatal factors	Placental abruption, cord compression, transplacental anesthetic or narcotic administration, intrauterine pneumonia, congenital cardiac anomalies, congenital pulmonary anomalies, birth trauma
Postnatal factors	Obstructed airway, maternal opioid exposure, congenital sepsis

In our infant, the initial lab findings and imaging were unremarkable. There were no significant maternal risk factors that would raise the suspicion of neonatal sepsis and there was a strong positive history of intrauterine exposure to hydromorphone two hours before delivery. This led to our high likelihood of suspicion of maternal narcotic administration as the cause of neonatal depression. An infant with IUGR is already at risk of having such depression [5]. The maternal narcotic administration might have further increased the risk of cardiorespiratory and neurological depression in our infant [4].

Parenteral opioids are frequently prescribed analgesia during labor [6]. Perinatal exposure to opioids four hours before delivery may be associated with neurological, cardiorespiratory depression, and feeding behavior problems in a newborn infant [7]. The effects of perinatal opioid exposure in a newborn infant largely depend upon the pharmacodynamics and pharmacokinetics of an opioid.

Hydromorphone is a semi-synthetic opioid agonist that is ten times more potent than morphine. It has higher lipid solubility and faster onset of action than morphine [8]. Hydromorphone readily crosses the placenta and the blood-brain barrier and binds to μ-opioid receptors in the central nervous system (CNS) of neonates resulting in rapid and pronounced neonatal depression [9]. Hydromorphone is metabolized by the liver enzyme uridyl glucuronosyltransferase 2B7 (UGT2B7) to a non-analgesic derivative. The maturation of UGT2B7 does not occur until six months of age. Thus, hydromorphone transferred from the mother to the baby may not be fully metabolized into a non-analgesic product. Hydromorphone might last for a longer duration than the expected terminal half-life of two to three hours in a newborn infant [8-10]. The pharmacokinetics of opioid is not well understood in IUGR infants. Due to the likelihood of immature glomerular filtration rate (GFR) and hepatic enzyme development, a larger volume of distribution, and decreased protein binding compared to the normal newborn infants, the duration of action and the clearance of hydromorphone is expected to be prolonged in an IUGR infant [11, 12]. Since hydromorphone is widely being used in obstetric analgesia, future studies on the pharmacodynamic and pharmacokinetic variances of hydromorphone in the normal-term newborn infant, IUGR infant, and preterm infants are needed. 

Naloxone is an opioid antagonist that blocks the effects of an opioid and reverses neonatal depression [2]. The Neonatal Resuscitation Program suggests naloxone should only be given to infants with severe respiratory depression after positive pressure ventilation has restored a normal heart rate and color and there is a history of maternal narcotic administration within the past four hours [13,14]. Maternal narcotic administration was two hours before delivery in our case and naloxone was given at eight hours of life. The elimination half-life of an intravenous dose of naloxone is approximately three hours in a normal-term newborn infant [15]. Thus, the dose of naloxone might need to be repeated based on the narcotic and the dose of narcotic to which the newborn has been exposed. The pharmacokinetics of naloxone has not been well studied in an IUGR infant. In our case, a single dose of naloxone was given, following which the infant showed immediate improvement, and there was no recurrence of signs and symptoms of neonatal depression. Hence the dose of naloxone was not repeated. Naloxone carries risks of precipitating acute withdrawal in an infant with chronic intrauterine exposure to opioids, and it might as well interfere with the normal functioning of the infant’s own natural opioids [2]. It is important as a physician to weigh the benefit versus risk of administering naloxone in a newborn infant. It is evident by our case that studies comparing the pharmacodynamic and pharmacokinetic variances of naloxone in normal-term newborn infants and IUGR infants are needed.

## Conclusions

This report describes a case of a term infant with IUGR exposed to hydromorphone two hours before delivery. The baby was apneic with poor tone stabilized with the initial resuscitative measures immediately after birth. At eight hours of life, the infant had respiratory and neurological depression possibly due to intrauterine exposure to hydromorphone reversed with a single dose of intravenous naloxone. Opioid exposure during labor might result in cardiorespiratory and neurological depression in a neonate. Although naloxone carries the risks of long-term side effects, naloxone might be considered for the reversal of persistent neonatal depression secondary to perinatal exposure to opioids within the four hours of delivery provided the mother is not narcotic dependent.
